# Cross-presentation of malaria antigen by brain microvessels: why CD8^+^ T cells are critical for the pathogenesis of experimental cerebral malaria

**DOI:** 10.1002/emmm.201302849

**Published:** 2013-06-05

**Authors:** Katsuyuki Yui

**Affiliations:** Division of Immunology, Department of Molecular Microbiology and Immunology, Nagasaki University, Graduate School of Biomedical SciencesNagasaki, Japan

**Keywords:** antigen presentation, brain, CD8^+^ T cells, malaria

See related article in EMBO Molecular Medicine http://dx.doi.org/10.1002/emmm.201202273

Cerebral malaria (CM) is one of the most serious complications of *Plasmodium falciparum* malaria. The disease mainly affects children under five in endemic areas and is the leading cause of death. Red blood cells (RBCs) infected with *P. falciparum* express parasite-derived molecules on the surface, which bind to host adhesion molecules expressed on vascular endothelial cells, leading to sequestration of infected RBCs in capillaries and microvessels. Pathogenesis of CM was previously thought to derive from the occlusion of brain microvessels induced by the sequestration of infected RBCs, but recent studies show that the etiology of CM is more complex and involves host immune responses (de Souza et al, 2010). Studies of human CM encounter difficulties due to ethical and local reasons, and histopathological examination is limited to post-mortem studies of fatal cases. Experimental cerebral malaria (ECM) in animal model is available, although there is a long-time debate regarding its usefulness as a model of human CM. Nevertheless, *Plasmodium berghei* ANKA (PbA) infection of susceptible mouse strains, such as C57BL/6 and CBA is the most widely used model where animals, develop the fatal disease (de Souza et al, 2010). These mice show clinical signs of neurological symptoms such as deviation of the head, ataxia and paralysis 6–12 days after infection, when the level of parasitaemia is relatively low, and often die shortly after the onset. The disease is characterized by the accumulation of infected RBCs and leukocytes in brain blood vessels and destruction of blood–brain barrier. Thus, ECM develops in a narrow time window after PbA infection in susceptible mice, suggesting that a combination of parasite infection and host immune responses culminate in the pathogenesis of ECM.

» Pathogenesis of CM was previously thought to derive from the occlusion of brain microvessels induced by the sequestration of infected RBCs, but recent studies show that the etiology of CM is more complex and involves host immune responses«

An interesting feature of ECM is the involvement of CD8^+^ T cells for its pathogenesis (Belnoue et al, 2002). Effector molecules of CD8^+^ T cells such as perforin and granzyme B are indispensable, indicating that cytolytic function of CD8^+^ T cells are directly involved in the pathogenesis of ECM (Nitcheu et al, 2003). Studies using recombinant malaria parasite expressing a model antigen demonstrated that PbA-specific CD8^+^ T cells are activated by dendritic cells (DCs) cross-presenting the malaria antigen during infection (Lundie et al, 2008; Miyakoda et al, 2008). It was also shown that vascular endothelial cells can acquire and cross-present exogenous antigens in association with MHC class I molecules (Valujskikh et al, 2002). Based on these observations, it was proposed that brain endothelial cells incorporate infected RBCs and cross-present malaria antigens in association with MHC class I molecules (Renia et al, 2006). *Plasmodium*-specific effector CD8^+^ T cells, which are activated by DCs, accumulate in brain blood vessels and directly attack these vascular endothelial cells. However, CD8 epitopes expressed in blood-stage malaria parasites were previously unknown.

**Figure 1:**
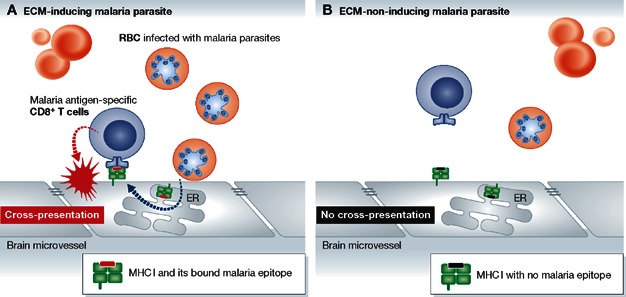
CD8^+^ T cells specific for malaria antigen accumulate in the brain of mice infected with ECM-inducing and non-inducing malaria parasites Brain microvessels incorporate malaria antigens and cross present them to specific CD8^+^ T cells, which deliver cytotoxic function and damage the microvessels.In mice infected with ECM-non-inducing strain of malaria parasites, brain microvessels do not cross-present malaria antigen, and do not become the target of CD8^+^ T cells.

In this issue, Howland et al shows that brain microvessels indeed cross-present *Plasmodium* antigens to CD8^+^ T cells (Howland et al, 2013). They identified predominant TCR αβ genes that are expressed in brain-sequestered CD8^+^ T cells. This TCR was used to define a major CD8 epitope of PbA, the glidesome-associated protein 50 (PbGAP50) and a MHC-tetramer, which can identify CD8^+^ T cells, was generated. Using this MHC-tetramer, the authors showed that a substantial proportion of effector CD8^+^ T cells in the infected mice recognize this PbGAP50. Interestingly, this epitope was also expressed in non-ECM inducing malaria parasites. A surprise came when they examined tetramer-positive cells in mice infected with the parasites; PbGAP50-specific CD8^+^ T cells were present in high proportions in the brain of mice infected with both PbA and non-ECM inducing parasites. In search for the mechanisms of ECM development in PbA-infected mice, they found that microvessel preparations from PbA-infected mice presented PbGAP50 peptide but not those from mice infected with non-ECM inducing strains (Fig 1). This study provides an explanation of why CD8^+^ T cells are critical for the pathogenesis of ECM. One caveat however remains, as the cell type that cross-presented malaria antigen has not been defined. Microvessels contain multiple cell types including endothelial cells, pericytes and astrocytes. While vascular endothelial cells are the primary candidates, purification and functional evaluation of each cell-type should be done.

» … microvessel preparations from PbA-infected mice presented PbGAP50 peptide but not those from mice infected with non-ECM inducing strains.«

This study also indicates that activation of *Plasmodium*-specific CD8^+^ T cells by DCs in periphery is not directly linked to ECM, and cross-presentation of malaria antigens by microvessel target cells, is critical for the pathogenesis. The authors explain that the difference between ECM-inducing and non-inducing parasites in microvessel cross-presentation comes from the amount of available antigen. The levels of infected RBCs attached to the brain microvessels of PbA-infected mice were much higher than those infected with non-ECM inducing strain. It remains to be answered, however, why PbA-infected RBCs preferentially accumulate in the brain. Is it due to the intrinsic properties of PbA or due to the immune response of the host? In addition, PbGAP50-specific CD8^+^ T cells alone did not reproduce ECM-like disease, which was induced only after treatment with enhancing reagent. Perhaps, multiple CD8 epitopes are involved in the pathogenesis of ECM, and it would be interesting to find other PbA antigens recognized by CD8^+^ T cells and ask whether their sum can induce an ECM-like disease. The other remaining question is why certain mouse strains are more or less susceptible to ECM, with C57BL/6 and CBA being highly susceptible (de Souza et al, 2010). This study is a significant advance for the understanding of the pathogenesis of ECM, but it seems that the whole picture of ECM pathogenesis is yet to be determined.

Finally, an important question is whether *Plasmodium*-specific CD8^+^ T cells are involved in the pathogenesis of human CM. It has been shown that malaria antigens can be transferred to human endothelial cells (Jambou et al, 2010). It would be interesting to know whether *Plasmodium*-specific CD8^+^ T cells are activated in malaria patients, and identify antigens of human malaria species that are recognized by CD8^+^ T cells. Identification of parasite antigen cross-presented by brain microvessels would be a significant advance towards understanding the pathogenesis of CM. Furthermore, it would be intriguing to speculate that mechanisms similar to ECM might underlie the pathogenesis of other human neurological encephalopathy.
